# Natural Convection and Irreversibility Evaluation in a Cubic Cavity with Partial Opening in Both Top and Bottom Sides

**DOI:** 10.3390/e21020116

**Published:** 2019-01-27

**Authors:** Hakan F. Oztop, Mohammed A. Almeshaal, Lioua Kolsi, Mohammed Mehdi Rashidi, Mohamed E. Ali

**Affiliations:** 1 Department of Mech. Engineering, Technology Faculty, Firat University, Elazig TR-23119, Turkey; 2 Department of Mechanical Engineering, College of Engineering, Al Imam Mohammad Ibn Saud Islamic University, Riyadh 11432, Saudi Arabia; 3 Department of Mech. Engineering, College of Engineering, Hail University, Hail 2240, Saudi Arabia; 4 Laboratoire de Métrologie et des Systèmes Énergétiques, École Nationale d’Ingénieurs, University of Monastir, Monastir 5000, Tunisia; 5 Shanghai Key Laboratory of Vehicle Aerodynamics and Vehicle Thermal Management Systems, Tongji University, Shanghai 200072, China; 6 ENN-Tongji Clean Energy Institute of Advanced Studies, Tongji University, Shanghai 200072, China; 7 Department of Mechanical Engineering, College of Engineering, King Saud University, Riyadh 11421, Saudi Arabia

**Keywords:** open cavity, natural convection, entropy, 3D flow

## Abstract

A numerical study on natural convection in a cubical cavity with partial top and bottom openings is performed in this paper. One of the vertical walls of the cavity has higher temperature than that of the opposite one; the remaining walls are insulated perfectly. Three-dimensional simulations of governing equations have been performed using a finite volume technique. The results are presented for different parameters such as opening length and Rayleigh number. It is observed that heat transfer rate and fluid flow can be controlled via opening ratio size and Rayleigh number.

## 1. Introduction

Convective heat transfer represents an important issue for various practical and engineering fields such as electronic cooling, solar heaters or building design. To make an efficient design, heat transfer and fluid flow are highly important parameters for these kinds of thermal systems.

Abib and Jaluria [1] computationally studied the free convection in cavities with partial openings. They solved the governing equation using the stream-function vorticity formulation based on the Boussinesq approximation. They noted that an increase of *Ra* causes a decrease in size of the recirculation cell that moves toward the vertical wall. Based on the Finite Volume Method (FVM), Polat and Bilgen [2] solved governing equation of natural convection in an opened tilted shallow cavitiy. Bilgen and Oztop [3] studied numerically the natural convection in an inclined cavity having partial opening. They observed that the inclination angle can be a means of control of heat transfer and flow structure inside the cavity. Bondareva et al. [4] used the heat-line visualization to analyze the flow structure and heat transfer in an open cavity filled with nanofluid and equipped with thick walls. Specifically, they solved a conjugate problem. Koufi et al. [5] studied numerically the mixed convection in open cavities. The authors considered the turbulent regime and tested the effects of the openings. Malekshah and Salari [6] performed both numerical and experimental analyses of free convection in cuboid geometries containing two immiscible fluids. Singh and Singh [7] investigated the combined radiation natural convection in inclined open cavities using the FVM. They concluded that the inclination is strongly determinant on heat transfer. Based on the Lattice Boltzmann Method (LBM), Sheikholeslami [8] investigated the hydrothermal behavior of the Magnetohydrodynamic (MHD) convection of nanofluid in an open cavity filled with porous media. He concluded that the rate of fluid exiting the cavity through the openings increases with Da and *Ra*. Bondareva et al. [9] used heat-line visualization to study the MHD flow in a wavy open cavity. Oztop et al. [10] considered three-dimensional partially open enclosures and performed a numerical investigation on three-dimensional natural convection with an evaluation of the different kinds of entropy generations.

Oztop et al. [11] studied the 2D natural convection in partially open cavities filled with porous media. They found that the increase of Grashof number increases the rate of heat transfer. Hinojosa et al. [12] analyzed the irreversibility generated by the natural convective flow and surface thermal radiation in a square open cavity. The surface thermal radiation was found to cause an important increase of the overall entropy generation. Hussain and Mustafa [13] studied the natural convection of a nanofluid in a locally heated parallelogrammic cavity having openings in its walls.

Singh and Singh [14] made a numerical solution on 2D natural convection in cavities with opened top wall considering the surface radiation. The effect of the volumetric heat generating source location on temperature field was studied. Correlations allowing the estimation of the maximum dimensionless temperature were proposed.

Bilgen and Muftuoglu [15] imposed a uniform heat flux on bouandries of open cavities. They found that heat transfer and the volume flow rates increases with *Ra*. Also, other studies related with open cavities and natural convection can be found in the literature, e.g., Prakash et al. [16], Gonzalez et al. [17] and Mohamad et al. [18]. Entropy generation can be calculated from the obtained data of temperature and velocities. It gives an opportunity to obtain energy losses inside the system. A numerical analysis of entropy production in an open cavity with pulsating flow in a horizontal channel has been presented by Zamzari et al. [19]. In a similar work, Mehrez et al. [20] performed work on entropy generation of nanofluids flow in an open cavity. Other works related to the subject can be found in [21,22,23,24,25,26,27,28,29,30,31,32,33].

In this work, a computational analysis on heat transfer, entropy generation and fluid flow due to natural convection in a cavity with partial opening from top and bottom sides is performed. Based on authors’ knowledge and the above literature, this work is a first step for the understanding of such 3D configuration.

## 2. Physical Model

**Figure 1:**
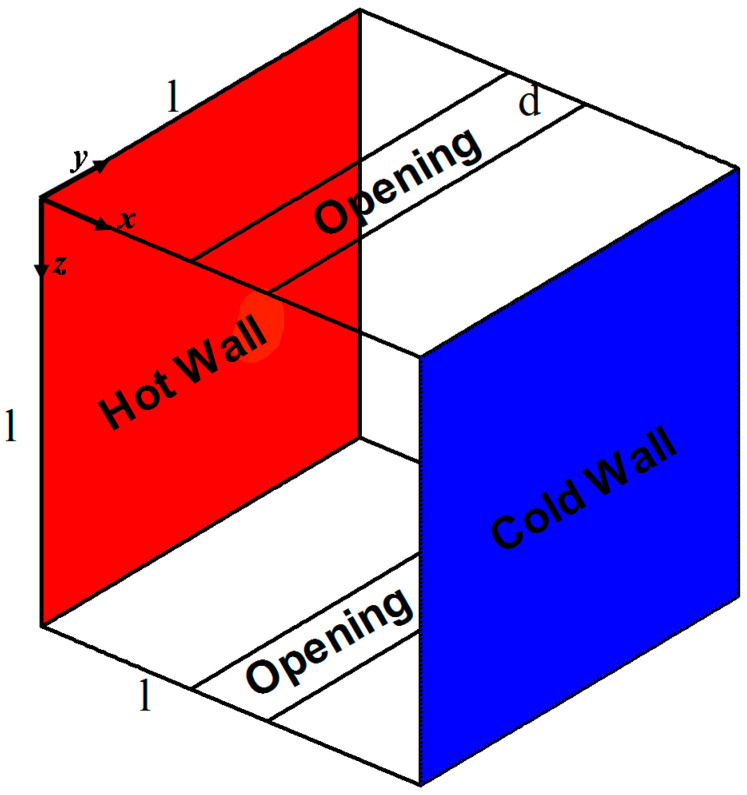
Physical model with boundary conditions and coordinates.

The studied configuration is presented with coordinates in Figure 1. The model consists of a cubical cavity having two openings, i.e., one each at the bottom and ceiling. The right and left walls are differentially heated, and all remaining walls are considered adiabatic. The fluid (air) is considered incompressible, and the Boussinesq approximation is considered.

## 3. Mathematical Formulation

The 3D formalism $\left(\vec{\psi }-\vec{\omega }\right)$ is used to simplify the treatment and the resolution of the equations governing the studied configuration by suppressing the pressure gradients terms. The vector potential ($\vec{\psi }$) and vorticity ($\vec{\omega }$) in 3D geometries are expressed by:

(1) $${\vec{\omega }}^{\prime }=\vec{\nabla }\times {\vec{V}}^{\prime }\;\mathrm{and}\;{\vec{V}}^{\prime }=\vec{\nabla }\times {\vec{\psi }}^{\prime }$$

In the dimensionless form, equations governing the phenomenon are:

(2) $$-\vec{\omega }=\nabla^{2}\vec{\psi }$$

(3) $$\frac{\partial \vec{\omega }}{\partial t}+(\vec{V}\times \nabla )\vec{\omega }-(\vec{\omega }\times \nabla )\vec{V}=\Delta \vec{\omega }+Ra\times Pr\times \left[\frac{\partial T}{\partial z};0;-\frac{\partial T}{\partial x}\right]$$

(4) $$\frac{\partial T}{\partial t}+\vec{V}\times \nabla T=\Delta T$$

The dimensionless numbers in the above equations can be expressed as:

(5) $$Pr=\frac{\nu }{\alpha }\;\mathrm{and}\;Ra=\frac{g\times \beta \times \Delta T\times {l^{\prime }}^{3}}{\nu \times \alpha }$$

**Boundary conditions**

The considered boundary conditions are as follows:

Temperature:

(6) T=1 at x=0

(7) T=0 at x=1

(8) $$\frac{\partial T}{\partial n}=0\;\mathrm{on}\;\mathrm{all}\;\mathrm{other}\;\mathrm{walls}$$

(9) $$\mathrm{At}\;\mathrm{open}\;\mathrm{boundary}:\;T_{in}=T_{c}\;\mathrm{if}\;n\times V<0\;\mathrm{and}\;{\frac{\partial T}{\partial n}|}_{out}=0\;\mathrm{if}\;n\times V\geq 0$$

Velocities

(10) $$V_{x}=V_{y}=V_{z}=0\;\mathrm{on}\;\mathrm{all}\;\mathrm{walls}$$

(11) $$\mathrm{At}\;\mathrm{open}\;\mathrm{boundaries}:\;\frac{\partial V_{x}}{\partial y}=\frac{\partial V_{y}}{\partial y}=\frac{\partial V_{z}}{\partial y}=0$$

Vorticities

(12) $$\omega_{x}=0,\;\omega_{y}=-\frac{\partial V_{z}}{\partial x},\;\omega_{z}=\frac{\partial V_{y}}{\partial x}\;\mathrm{at}\;x=0\;\mathrm{and}\;1$$

(13) $$\omega_{x}=\frac{\partial V_{z}}{\partial y},\omega_{y}=0,\;\omega_{z}=-\frac{\partial V_{x}}{\partial y}\;\mathrm{at}\;y=0\;\mathrm{and}\;1$$

(14) $$\omega_{x}=-\frac{\partial V_{y}}{\partial z},\;\omega_{y}=\frac{\partial V_{x}}{\partial z},\;\omega_{z}=0\;\mathrm{at}\;z=0\;\mathrm{and}\;1$$

Vector potential:

(15) $$\frac{\partial \psi_{x}}{\partial x}=\psi_{y}=\psi_{z}=0\;\mathrm{at}\;x=0\;\mathrm{and}\;1$$

(16) $$\psi_{x}=\frac{\partial \psi_{y}}{\partial y}=\psi_{z}=0\;\mathrm{at}\;y=0\;\mathrm{and}\;1$$

(17) $$\psi_{x}=\psi_{y}=\frac{\partial \psi_{z}}{\partial z}=0\;\mathrm{at}\;z=0\;\mathrm{and}\;1$$

The generated entropy is expressed by:

(18) $${S^{\prime }}_{gen}=-\frac{1}{{T^{\prime }}^{2}}\times \vec{q}\times \vec{\nabla }T^{\prime }+\frac{\mu }{T^{\prime }}\times \varphi^{\prime }$$

With:

(19) $$\phi^{\prime }=2\left[{\left(\frac{\partial {V^{\prime }}_{x}}{\partial x^{\prime }}\right)}^{2}+{\left(\frac{\partial {V^{\prime }}_{y}}{\partial y^{\prime }}\right)}^{2}+{\left(\frac{\partial {V^{\prime }}_{z}}{\partial z^{\prime }}\right)}^{2}\right]+{\left(\frac{\partial {V^{\prime }}_{y}}{\partial x^{\prime }}+\frac{\partial {V^{\prime }}_{x}}{\partial y^{\prime }}\right)}^{2}+{\left(\frac{\partial {V^{\prime }}_{z}}{\partial y^{\prime }}+\frac{\partial {V^{\prime }}_{y}}{\partial z^{\prime }}\right)}^{2}+{\left(\frac{\partial {V^{\prime }}_{x}}{\partial z^{\prime }}+\frac{\partial {V^{\prime }}_{z}}{\partial x^{\prime }}\right)}^{2}$$

Thus:

(20) $${S^{\prime }}_{gen}=\frac{k}{{T^{\prime }}_{0}^{2}}\left[{\left(\frac{\partial T^{\prime }}{\partial x^{\prime }}\right)}^{2}+{\left(\frac{\partial T^{\prime }}{\partial y^{\prime }}\right)}^{2}+{\left(\frac{\partial T^{\prime }}{\partial z^{\prime }}\right)}^{2}\right]+\frac{\mu }{T_{0}}\left\{\begin{array}{l} 2\times \left[{\left(\frac{\partial {V^{\prime }}_{x}}{\partial x^{\prime }}\right)}^{2}+{\left(\frac{\partial {V^{\prime }}_{y}}{\partial y^{\prime }}\right)}^{2}+{\left(\frac{\partial {V^{\prime }}_{z}}{\partial z^{\prime }}\right)}^{2}\right]\\ +{\left(\frac{\partial {V^{\prime }}_{y}}{\partial x^{\prime }}+\frac{\partial {V^{\prime }}_{x}}{\partial y^{\prime }}\right)}^{2}+{\left(\frac{\partial {V^{\prime }}_{z}}{\partial y^{\prime }}+\frac{\partial {V^{\prime }}_{y}}{\partial z^{\prime }}\right)}^{2}+{\left(\frac{\partial {V^{\prime }}_{x}}{\partial z^{\prime }}+\frac{\partial {V^{\prime }}_{z}}{\partial x^{\prime }}\right)}^{2} \end{array}\right\}$$

The dimensionless local entropy generation is expressed as:

(21) $$N_{s}={S^{\prime }}_{gen}\frac{1}{k}{\left(\frac{lT_{0}}{\Delta T}\right)}^{2}$$

(22) $$\begin{array}{l} N_{s}=\left[{\left(\frac{\partial T}{\partial x}\right)}^{2}+{\left(\frac{\partial T}{\partial y}\right)}^{2}+{\left(\frac{\partial T}{\partial z}\right)}^{2}\right]\\ +\phi \times \left\{2\left[{\left(\frac{\partial V_{x}}{\partial x}\right)}^{2}+{\left(\frac{\partial V_{y}}{\partial y}\right)}^{2}+{\left(\frac{\partial V_{z}}{\partial z}\right)}^{2}\right]+\left[{\left(\frac{\partial V_{y}}{\partial x}+\frac{\partial V_{x}}{\partial y}\right)}^{2}+{\left(\frac{\partial V_{z}}{\partial y}+\frac{\partial V_{y}}{\partial z}\right)}^{2}+{\left(\frac{\partial V_{x}}{\partial z}+\frac{\partial V_{z}}{\partial x}\right)}^{2}\right]\right\} \end{array}$$

with $\phi =\frac{\mu \alpha^{2}T_{m}}{l^{2}k\Delta T^{2}}$ is the irreversibility coefficient.

The total dimensionless entropy generation is:

(23) $$S_{tot}=\int_{v}N_{s}dv=\int_{v}\left(N_{s-th}+N_{s-fr}\right)dv=S_{th}+S_{fr}$$

Average Bejan number (*Be*) is evaluated using the following expression:

(24) $$Be=\frac{S_{th}}{S_{th}+S_{fr}+S_{J}}$$

Local and average Nusselt numbers are expressed respectively using:

(25) $$Nu={\frac{\partial T}{\partial x}|}_{x=0}\;\mathrm{and}\;Nu_{av}=\int_{0}^{1}\int_{0}^{1}Nu\;dy\;dz$$

The solutions of the above described governing equations were obtained using a code developed in the FORTRAN language. The FVM is used to develop the governing equations, the convective terms and temporal derivatives are discretized via the central-difference scheme and the fully implicit procedure, respectively. The solution is considered satisfactory if:

(26) $$\sum_{i}^{1,2,3}\frac{\max\left|\psi_{i}^{n}-\psi_{i}^{n-1}\right|}{\max\left|\psi_{i}^{n}\right|}+\max\left|T_{i}^{n}-T_{i}^{n-1}\right|\leq 10^{-4}$$

## 4. Verification and Grid Sensitive Study

**Figure 2:**
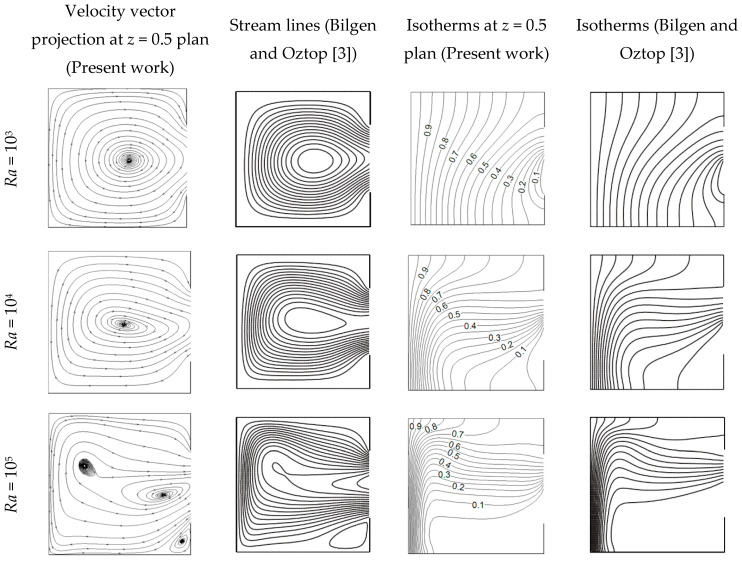
Comparison with results of Bilgen and Oztop [3].

As first verification the results of the present code have been compared with the 2D results of Bilgen and Oztop [3]. As shown in Figure 2, a good concordance in the flow structure and temperature field is encountered. A second verification based on 3D works of Wakashima and Saitho [34] for air filled cubic cavity is conducted and presented in Table 1. It can be concluded from the table that the code gives satisfactory results compared with those presented in the literature.

The grid sensitivity test has been performed for *Pr* = 0.7, *Ra* = 10^5^ and *d* = 0.5. The average Nusselt number and the maximum of *x*-component velocity were used as testing parameters. Four spatial meshes of 61^3^, 71^3^, 81^3^ and 91^3^ were compared. The results of the grid dependency test are presented in Table 2. The increases from the grid 81^3^ to 91^3^ are 0.149% for *Nu_av_* and 0.734% for $V_{x\max}$. Thus, for computational economy and accuracy, spatial mesh size of 81^3^ and time step of 10^−4^ are chosen to perform all simulations in the present work.

## 5. Results and Discussion

A numerical study is performed to investigate the natural convection heat transfer and fluid flow in a cavity with partially opened top and bottom sides. The effects of *Ra* and the dimension of the opening are highlighted. 

**Figure 3:**
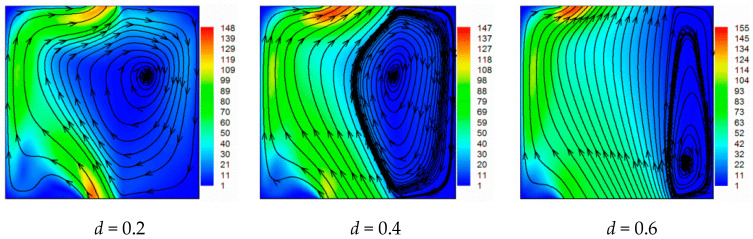

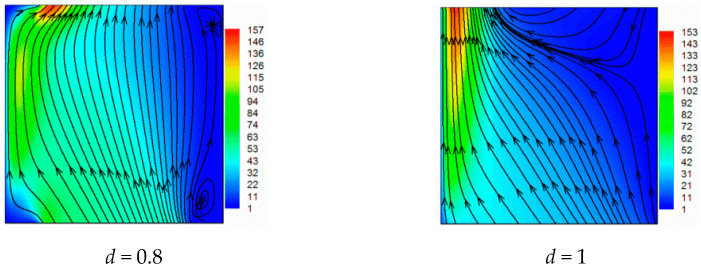
Velocity vectors projections and magnitude of velocity at *z* = 0.5 plan for different opening widths and *Ra* = 10^5^.

Figure 3 illustrates the velocity vector projections and magnitude of velocity at *z* = 0.5 for *Ra* = 10^5^ and different opening ratios. For *d* = 0.2, a huge vortex is located on the right side. The size of this clockwise circulating cell is reduced for *d* = 0.4. It becomes smaller and is pushed to right bottom side for *d* = 0.6. For *d* = 0.8 the cell. The flow goes from the bottom to the top opening, and lines are almost parallel to vertical walls with the very small circulating cell becoming very small. For *d* = 1, the circulation cell disappears and the flow passes directly from the bottom to the top of the cavity, except at the top right region, where the fluid enters and leaves from the top opening. It is interesting to note that flow comes into the cavity from the top side and dissipates suddenly for fully opened cases. In all cases, no symmetric structure was encountered.

**Figure 4:**
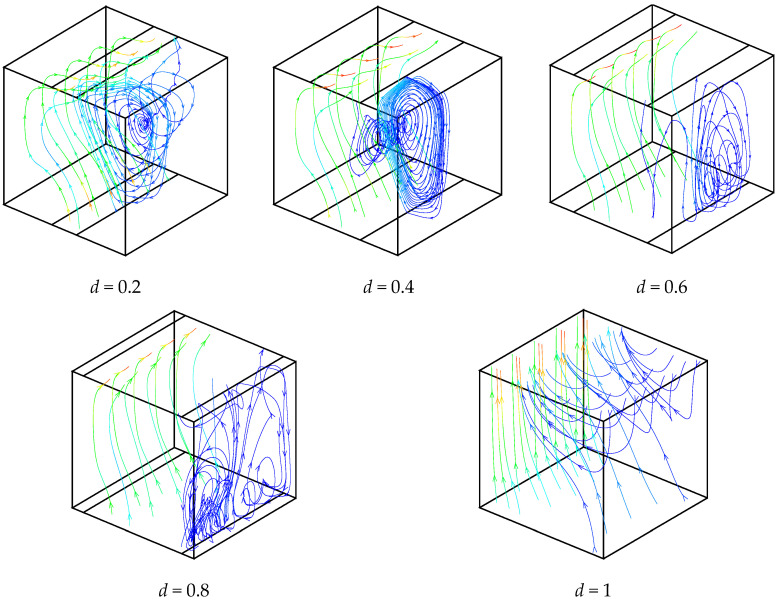
Particles trajectories for different opening widths and *Ra* = 10^5^.

In order to show the 3D structure of the flow, Figure 4 illustrates some particles trajectories for different opening widths at *Ra* = 10^5^. The obtained results are compared with a fully opened case. As seen from the figure, the opening ratio directly effects the circulation cell dimension and location. For a fully opened case, no circulation cell is formed inside the cavity. This is an interesting result; flow does not have any circulation near the hot side for all cases due to a higher flow velocity. Due to the existence of circulation vortexes (for *d* < 1), the fluid passes transversally through the enclosure, which represents a purely 3D characteristic. For all presented cases, the circulation vortexes are convergent from the front and back walls to the center of the cavity.

**Figure 5:**
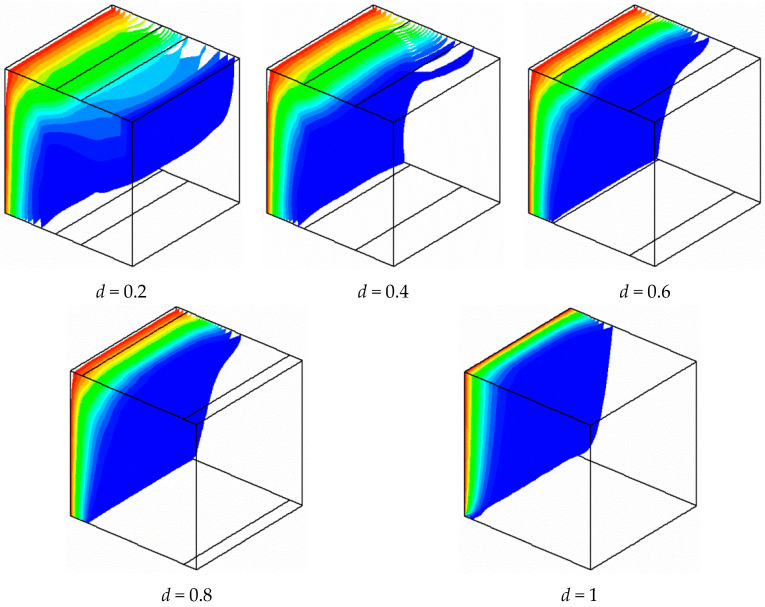
Temperature field for different opening widths and *Ra* = 10^5^.

As seen from the results of the temperature fields presented in Figure 5, iso-surfaces of temperature are pushed to the heated wall with increases in the opening ratio. This result is due to the increase of the flow velocity created by the presence of the openings. For *d* = 0.2, vertical stratification exists in the central region of the cavity due to the great size of the circulation vortex. By increasing the openings size, the vertical stratification disappears and the iso-surfaces of temperature become more piled near the hot wall due to the disappearance of the recirculation vortexes caused by the direct flow between the bottom and top openings.

**Figure 6:**
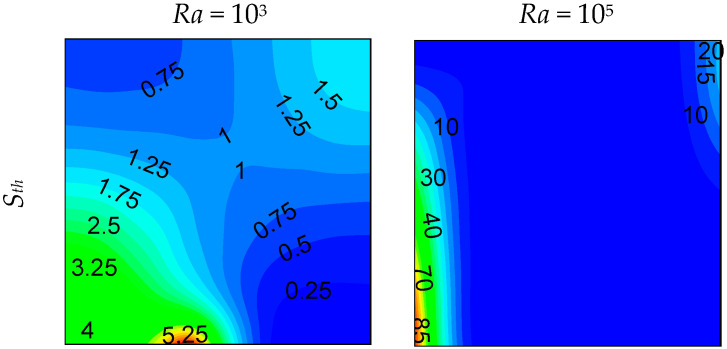

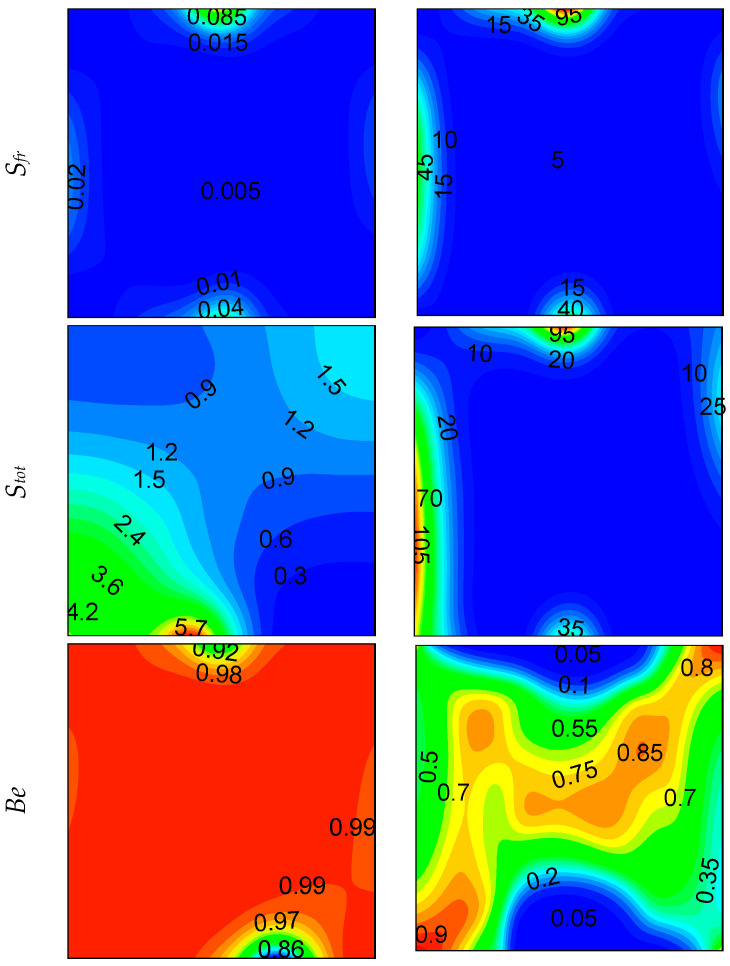
Local entropy generations at (*z* = 0.5) plan, for *d* = 0.1.

**Figure 7:**
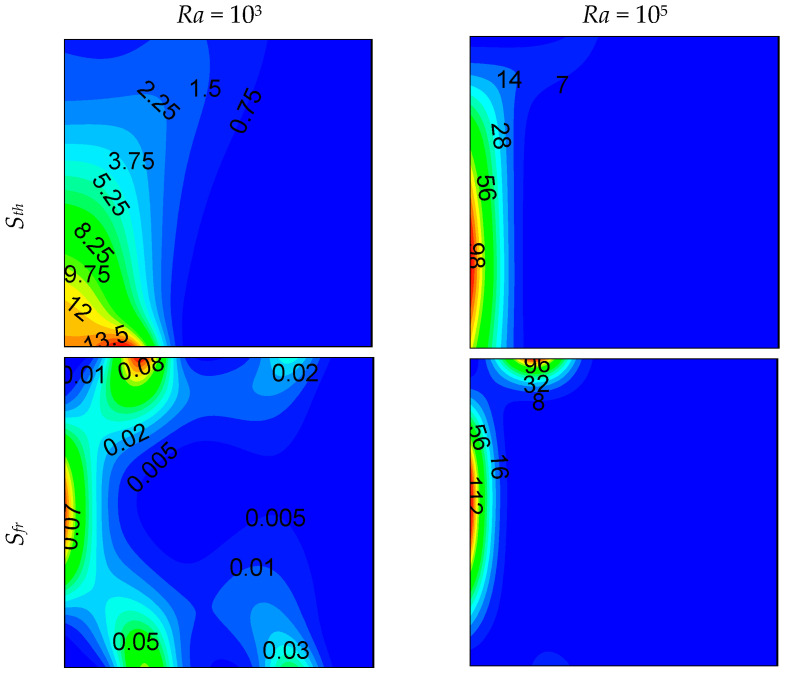

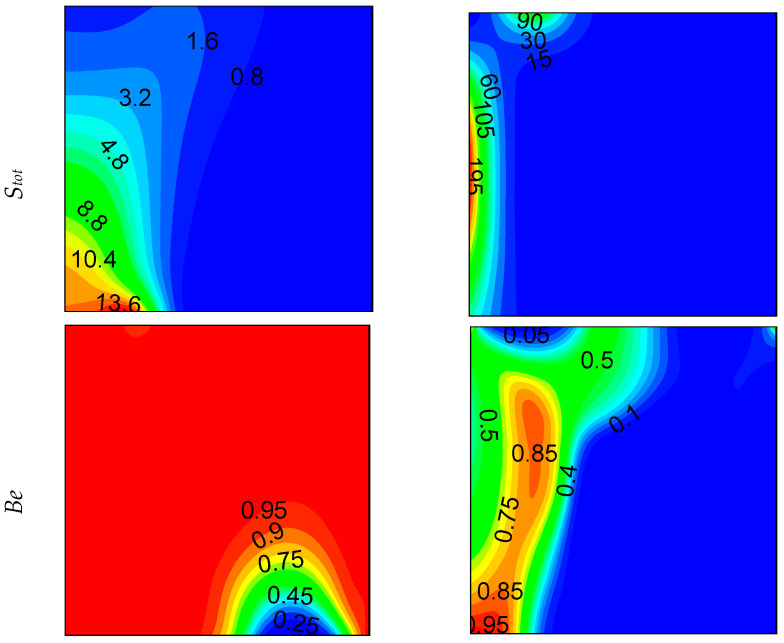
Local entropy generations at (*z* = 0.5) plan, for *d* = 0.5.

**Figure 8:**
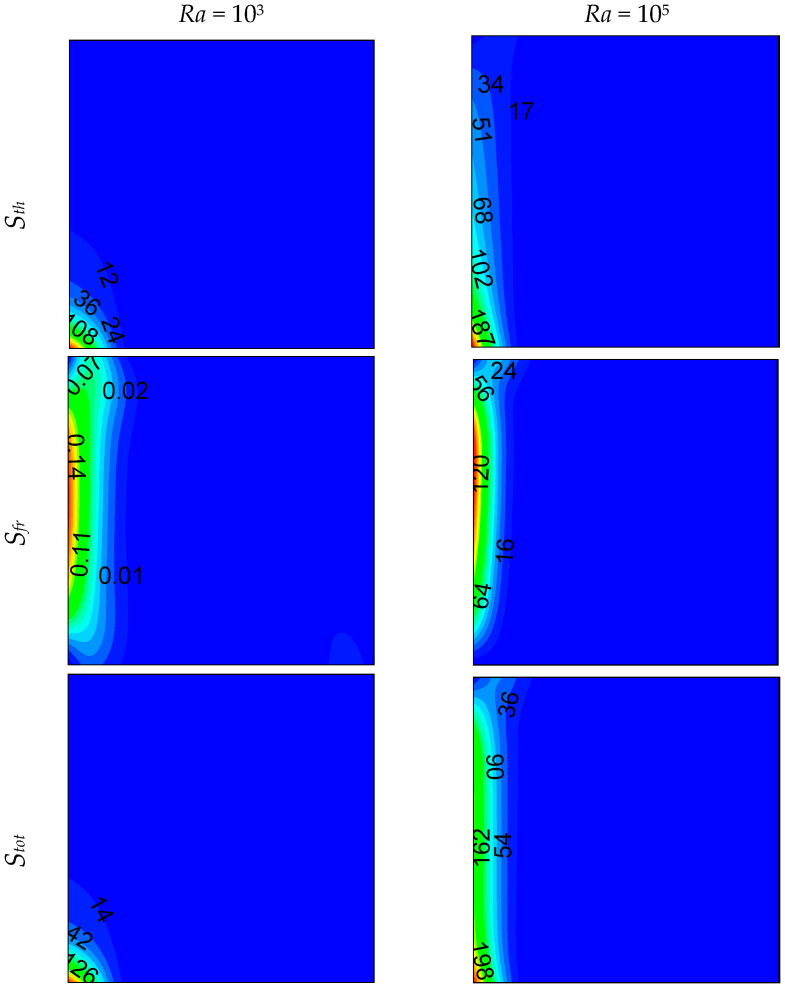

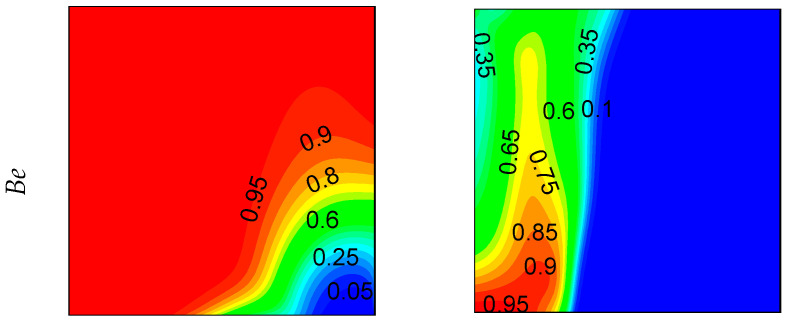
Local entropy generations at (*z* = 0.5) plan, for *d* = 0.9.

Local entropy-generated contours at *z* = 0.5 plan are shown in Figure 6, Figure 7 and Figure 8 for different opening sizes and different Rayleigh numbers. For low Rayleigh numbers and small opening ratios, thermal entropy generation (*S_th_*) is distributed across the entire domain. By increasing the Rayleigh number, *S_th_* becomes concentrated near the hot wall, and by increasing *d*, it becomes concentrated near the bottom right corner due to the increase of temperature gradients in this region, as shown in Figure 5. In contrast to thermal entropy generation, frictional irreversibility becomes distributed across the entire cavity by increasing the opening ratio due to the enlargement of the domain from where the flow passes from the bottom to the top opening, causing an intensification of the flow, and consequently, an increase of the fluid-fluid friction. In all cases, the total entropy profile is similar to that of the thermal one, showing a dominance of *S_th_* compared to *S_fr_*, especially for low Rayleigh numbers, where *S_th_* dominates in the domain. For higher *Ra*, the dominance is more pronounced on the right side of the cavity. This ascertainment is boosted by the Bejan number profile. 

**Figure 9:**
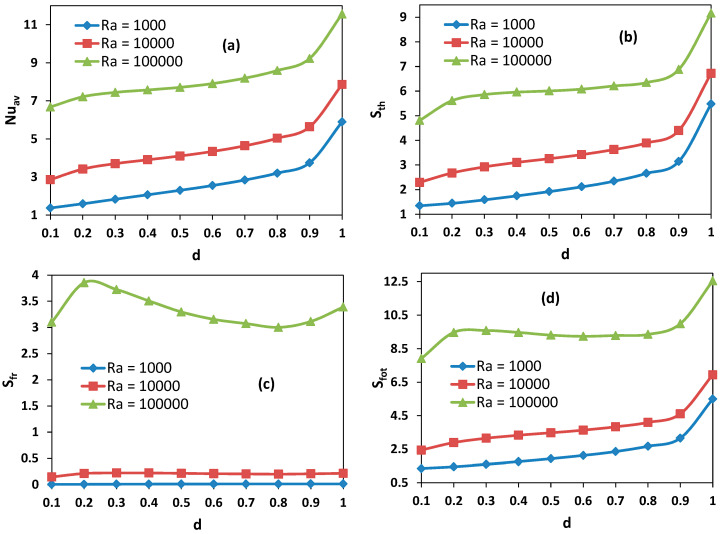

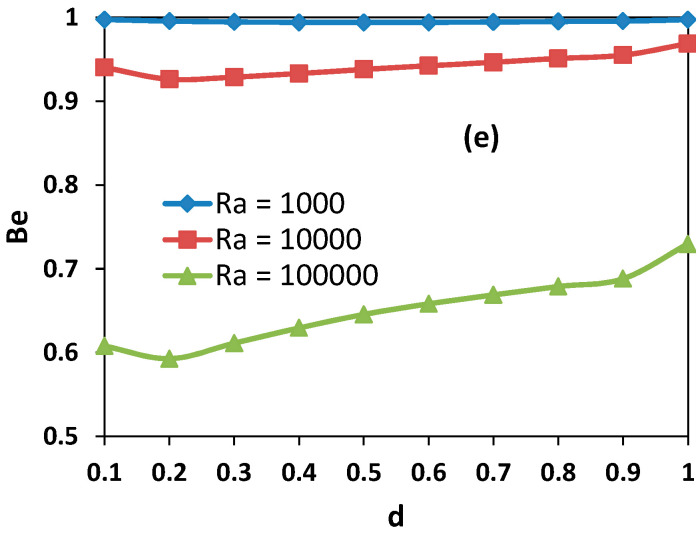
Effect of opening size on (**a**) average Nusselt number, (**b**) Thermal entropy generation, (**c**) Viscous entropy generation, (**d**) Total entropy generation, (**e**) Bejan number.

Figure 9a–e present the variations of average Nusselt number, thermal, viscous and total entropy generations and Bejan number with opening ratio. It is observed that higher heat transfer occurs for higher values of *Ra*, and that heat transfer increases almost linearly with opening ratio. This increment is clear for the lowest value of the Rayleigh number. Similarly, thermal entropy generation increases with both Rayleigh numbers and opening ratio almost linearly. But there is a huge increment for fully opened cavities. 

The opening ratio becomes insignificant on viscous entropy generation for lower values of Rayleigh number. In contrast, a wavy distribution is formed for entropy generation due to fluid friction versus opening ratio. It is an interesting that there is a maximum value around *d* = 0.2. This result can serve for optimization purposes. The variation of total entropy generation shows a similar trend with thermal entropy generation except for high *Ra* and low opening sizes. The Bejan number is almost constant with opening ratio for lower Rayleigh numbers, and is around of 1, showing the dominance of thermal entropy generation. In contrast, a linear increase occurs for high Rayleigh numbers.

## 6. Conclusions

A numerical study has been performed to examine the heat transfer and fluid flow in a cubical cavity with top and bottom openings. The main findings can be listed as

- Heat transfer enhances with increasing the opening ratios and Rayleigh numbers.
- The flow field can be controlled in a cavity with the width of opening part. Both the dimension and location of circulation cells can be controlled.
- The novelty of this work was to undertake an analysis of the natural cooling and natural ventilation problem of a model room. Thus, the results can be used for building ventilation.
- As expected, the flow is more pronounced near of heated part compared to other regions.
- The obtained results can be used for some kinds of filters or heating and cooling systems.
- Increasing the opening part increases the entropy generation almost linearly for lower values of the Rayleigh number.

## Figures and Tables

**Table 1:** Comparison with results of Wakashima and Saitho [34] (presented between parentheses).

*Ra*	$\psi_{z}$ (center)	$\omega_{z}$ (center)	$Nu_{av}$
10^4^	0.05528 (0.05492)	1.1063 (1.1018)	2.062 (2.062)
10^5^	0.034 (0.03403)	0.262 (0.2573)	4.378 (4.366)
10^6^	0.01972 (0.01976)	0.1284 (0.1366)	8.618 (8.6097)

**Table 2:** Grid sensitivity test for *Pr* = 0.7, *Ra* = 10^5^, and *d* = 0.5.

Grid	*Nu_avg_*	$V_{x\max}$
61^3^	7.62	135.442
71^3^	7.6571	137.565
81^3^	7.71246	141.871
91^3^	7.724	142.913

## Nomenclature

Cp	Specific heat at constant pressure (J/kg·K)
*d*	Dimensionless opening width
*g*	Gravitational acceleration (m/s^2^)
*h*	Dimensionless opening length, *h*’/*l*’
*k*	Thermal conductivity (W/m·K)
*l*	Dimensionless cavity width
*Nu*	Local Nusselt number
*Nu_av_*	Average Nusselt number
*Pr*	Prandtl number
*Ra*	Rayleigh number
*t*	Dimensionless time ($t^{\prime }\times \alpha /l^{2}$)
*T*	Dimensionless temperature [$\left(T^{\prime }-{T^{\prime }}_{c})/({T^{\prime }}_{h}-{T^{\prime }}_{c})\right]$
${T^{\prime }}_{c}$	Cold temperature (K)
${T^{\prime }}_{h}$	Hot temperature (K)
$\vec{V}$	Dimensionless velocity vector (${\vec{V}}^{\prime }\cdot l/\alpha$)
*x*, *y*, *z*	Dimensionless Cartesian coordinates ($x^{\prime }/l$, $y^{\prime }/l$, $z^{\prime }/l$)
**Greek Symbols**	
α	Thermal diffusivity (m^2^/s)
β	Thermal expansion coefficient (1/K)
ρ	Density (kg/m^3^)
μ	Dynamic viscosity (kg/m·s)
ν	Kinematic viscosity (m^2^/s)
$\vec{\psi }$	Dimensionless vector potential ($\vec{\psi }\prime /\alpha$)
$\vec{\omega }$	Dimensionless vorticity (${\vec{\omega }}^{\prime }\times \alpha /l^{2}$)
ΔT	Dimensionless temperature difference
**Subscripts**	
av	Average
*x*, *y*, *z*	Cartesian coordinates
